# Dissociative Amnesia: A Mist Over Psychosis

**DOI:** 10.7759/cureus.44619

**Published:** 2023-09-03

**Authors:** Bashayer M Almaazmi, Abdullah N Aldweik, Mazin A Mukhtar

**Affiliations:** 1 Psychiatry, Al Amal Psychiatric Hospital, Dubai, ARE; 2 Medicine, University of Sharjah, Sharjah, ARE

**Keywords:** dissociation, affective disorder, psychotic disorder, psychosis, brief psychotic disorder, dissociative amnesia

## Abstract

This case report highlights the unique presentation of dissociative amnesia masking an underlying brief psychotic disorder, triggered by a very intense psycho-social stressor. Learning points from this report center around the importance of considering psychotic as well as affective disorders alongside a presentation of dissociative amnesia, and not only the expected anxious, post-traumatic or personality-oriented states.

Our patient, a 37-year-old gentleman, was brought to our emergency department via police referral. He had gaps in his autobiographical memory that, upon receiving a regular dose of benzodiazepines, unraveled bizarre, uncooperative, and agitated behavior as well as marked fluctuations in his daily mental state examinations. Biological management through antipsychotic monotherapy, psychological management through insight-oriented therapy as well as psychological support, and social management revolving around the alleviation of surrounding stressors enabled his safe recovery.

## Introduction

Dissociative amnesia is a condition in which an individual experiences memory loss and is unable to recall autobiographical information. It most commonly presents with retrograde memory loss rather than anterograde memory loss and this forgetfulness is not better explained by, or attributed to, injury to the brain or any neurological dysfunction, nor is it consistent with ordinary forgetfulness [1]. The usual pretext is a significant stressor or traumatic event with frequent co-morbid conditions being personality disorders, anxiety disorders as well as psychotic disorders [2,3].

Though its prevalence is not well-established, dissociative amnesia is noted to have a prevalence of 1.8%, often diagnosed within the age group of 20 to 40 years old [4]. There are different sub-categories of dissociative amnesia. There is localized amnesia: being unable to recall specific events; selective amnesia: forgetting only parts of the traumatic event and can occur alongside localized amnesia; generalized amnesia: the forgetting of complete life histories; systematized amnesia: where patients can forget all information within a specific category; and continuous amnesia: where patients forget each new event as it occurs. Rarely, dissociative amnesia occurs with fugue. In such instances, the individual not only experiences loss in memory but a sense of identity as well, allowing them to take on a new persona and live a completely different life from the one they had lived previously [5].

Differential diagnoses that need to be ruled out include those of neurological origin, such as head injuries, brain lesions, brain tumors, brain inflammation, and seizures. It is of essence to rule out any substance use. It is also important to bear in mind various psychiatric entities such as post-traumatic stress disorder, somatic symptom disorder, conversion disorder as well as manifestations of personality disorders [6].

The duration of which the amnesic episode would last is undetermined and curtailed to the patient experience. Management is personalized but includes pharmacological administration of benzodiazepines, as well as psychotherapeutic interventions such as cognitive behavioral therapy in order to alleviate anxieties and untangle the underlying trauma. However, noted elimination of an identifying precipitating factor has been seen to improve prognosis, highlighting the importance of psychological and social support [7].

## Case presentation

This case study explores the case of a 37-year-old gentleman, who was brought to our emergency department via police referral due to “abnormal behavior,” with no further collateral provided. Initial assessment proved difficult as the patient was uncooperative, refusing to answer questions; however, there was no noted agitation or aggression in his presentation. However, his subsequent interviews with the team in the psychiatric unit revealed gaps in our patient's memory, on a background of a sudden and severe stressor in the workplace. 

The majority of our patient's memory loss pertained to autobiographic information, with our patient being unable to recall his place of work, his family, or close contacts. He was started on lorazepam 1 mg PO BID and showed notable improvement as he began to superficially elaborate, in subsequent interviews, about his personal history. Our patient reported that he was recently employed as a marine engineer where he was not only overwhelmed by the intense workload but also clashed with colleagues, resulting in a bout of irritability and aggression that, two days ago, led to physical violence. After that, he had been on the streets; however, he did not recall being collected by the police and being brought to hospital. He denied any past psychiatric history, substance abuse, or any familial psychiatric history. He denied the presence of psychotic symptoms, then and now, and no manic symptoms could be observed at the time. This was then confirmed by a collateral history taken from our patient's friend, currently living in the country with him, and his mother, currently living abroad.

**Figure 1:**
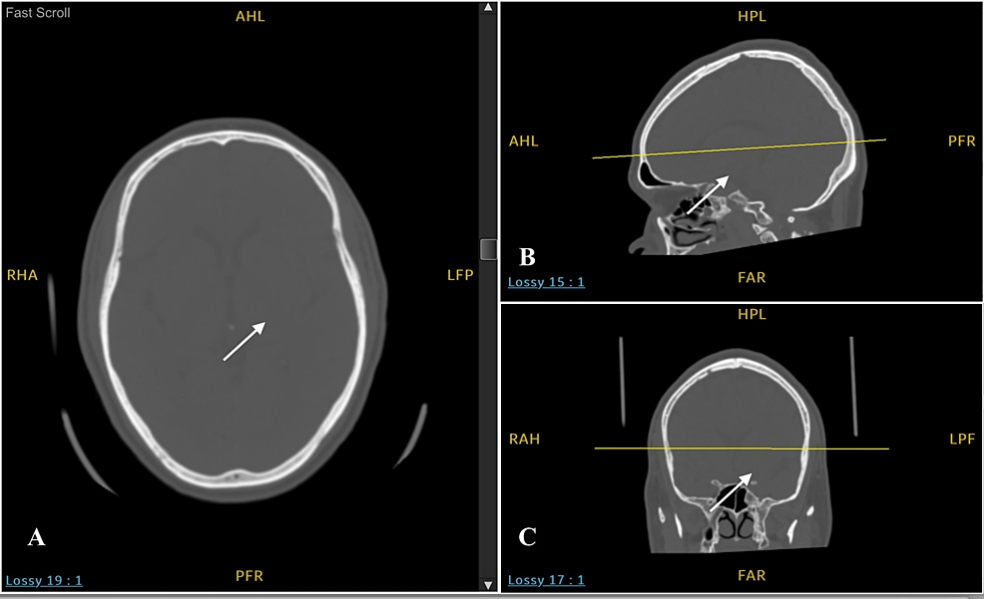
CT brain without contrast in the (A) axial (B) sagittal and (C) coronal planes showing (arrow) old infarcts in the left basal ganglia.

In order to rule out organic causes, a full set of investigations were placed. Complete blood count, renal function tests, and liver function tests were within normal limits and his urine drug screen was negative for any substances. The patient denied a history of seizures as well as a family history of epilepsy. A consultation with neurology was placed. Their recommendation of a CT brain without contrast (Figure 1) revealed old infarcts in the left basal ganglia. This allowed us to rule out the presence of any tumors, lesions, or inflammation within the brain. 

During his stay, the team noticed fluctuations in the patient’s mood, behavior, and memory. He sustained a well-groomed appearance, his speech generally being within acceptable parameters of tone, volume, and rate. When it came to his mood, there were days when our patient would be amiable, pleasant, and cooperative, reporting a fair mood and displaying a congruent, reactive affect. But, there were days when he would be irritable, refusing to answer questions and, at times, closing his eyes and sitting silently in the interview room. He was also noted to have bouts of grandiosity and a sense of inflated self-esteem. When it came to his behavior, there were moments where he was noted to behave bizarrely: running across table tops, hoarding the wheelchair of his fellow patient, restlessly pacing, and reporting incoherently to nursing staff. When our patient was confronted regarding these incidents, he stated that he did not recall acting in such a way. A notable incident occurred when he entered the interview room and became very irritable upon seeing the team, as he did not recognize them as his treating team and demanded their presence. 

He was commended on olanzapine, eventually reaching 15 mg PO bedtime, with striking improvement in his mood, behavior, and his memory. Insight-oriented therapy, as well as psycho-education on his presentation, current stay, and expected outcome were done by the team, with dissociative amnesia being diagnosed alongside brief psychotic disorder, as it explained the mélange of signs and symptoms existing under the fog of dissociation, coming to light over the course of his stay.

As an inpatient, he was under our care for exactly 30 days. After discharge, we organized a follow-up via phone call within a week as well as a face-to-face appointment in the outpatient clinic within a month. 

## Discussion

Dissociative amnesia is known by its definition: the inability to recall important autobiographic information that would usually be readily remembered, of which could be seen with our patient. However, what’s worth discussing is the spectrum of mental illness that can co-exist alongside or linger beneath the haze of dissociative amnesia. It is well-known that dissociative amnesia occurs in a background history of patients with post-traumatic stress disorder, somatization, conversion disorder as well as personality disorders, but little discussion is made on the cohort of patients that are on the psychotic or the affective spectrum [8]. At times, this can bring professional doubt, as an unfamiliar occurrence in a setting of pattern recognition can, at times, make physicians think twice before donning the diagnosis and, thereby, the optimal treatment plan. However, in our experience with our patient, giving him the chance to settle with benzodiazepines allowed the gentle easing of his dissociative amnesia, bringing to light a constellation of symptoms curated by his recent stressors. 

A meta-analysis by Longden et al., 2020 explored the relationship between dissociative symptoms and psychosis, using the DSM-V definition as a guideline [9]. That is, dissociation is “a disruption of and/or discontinuity in the normal integration of consciousness, memory, identity, emotion, perception, body representation, motor control and behavior.” Their results underlined the fact that dissociation and psychosis are related to multiple positive symptoms and are related to higher patient disorganization. Interestingly, such behavior was witnessed with our patient who exhibited a range of bizarre behavior that alluded to his psychotic disorder. 

The importance of psychiatric management goes beyond the acute phase, and, with dissociative amnesia, there is an emphasis on alleviating stressors as well as giving meaning to the episode experienced. Our patient had recently immigrated to the country with no family or support system and, the company at which he worked as a marine engineer had only recently employed him. As per our patient, he had been overwhelmed by the job; however, collaterals also highlighted that an incident had occurred at work that cost the company damages and, hence, our patient faced imminent termination. An escalation of troubles coming to a peak, triggering a reaction. Within our management, we included reaching out to those who could support him, finding a friend who worked in a nearby city. That enabled supervision post-discharge. As for his career, we held lengthy discussions regarding his eventual return to his home country as well as the processing of his travel documents, allowing the mapping of a plan. A step into certainty and out of the haze of dissociation [10]. 

## Conclusions

Our case explores the unraveling of psychosis after the dissipation of dissociative amnesia. Our patient presented with memory gaps pertaining to autobiographical information that, after a course of waxing and waning, revealed the disorganized behavior of a psychotic disorder. Through this case, we can appreciate the importance of considering co-morbid psychotic psychiatric conditions alongside a presentation of memory loss.
